# Non-invasive sources of cells with primary cilia from pediatric and adult patients

**DOI:** 10.1186/s13630-015-0017-x

**Published:** 2015-06-01

**Authors:** Henry Ajzenberg, Gisela G. Slaats, Marijn F. Stokman, Heleen H. Arts, Ive Logister, Hester Y. Kroes, Kirsten Y. Renkema, Mieke M. van Haelst, Paulien A. Terhal, Iris A. van Rooij, Mandy G. Keijzer-Veen, Nine V. Knoers, Marc R. Lilien, Michael A. Jewett, Rachel H. Giles

**Affiliations:** Department of Nephrology and Hypertension, University Medical Center Utrecht, Heidelberglaan 100, 3584CX Utrecht, Netherlands; Department of Medical Genetics, University Medical Center Utrecht, Lundlaan 6, 3584EA Utrecht, Netherlands; Department of Human Genetics, Radboud University Medical Center, Geert Grooteplein zuid 10, 6525GA Nijmegen, Netherlands; Department of Health Evidence, Radboud University Medical Center, Geert Grooteplein zuid 10, 6525GA Nijmegen, Netherlands; Department of Pediatric Nephrology, University Medical Center Utrecht, Lundlaan 6, 3584EA Utrecht, Netherlands; Department of Surgery (Urology), University of Toronto, 610 University Avenue, M5G 2M9 Toronto, Canada; Department of Biochemistry, University of Western Ontario, Medical Sciences Building Rm. 342, N6A 5C1 London Ontario, Canada

**Keywords:** Pediatrics, Urine, Deciduous tooth, Cell culture, Protocol, Cilia, Ciliopathy

## Abstract

**Background:**

Ciliopathies give rise to a multitude of organ-specific pathologies; obtaining relevant primary patient material is useful for both diagnostics and research. However, acquisition of primary ciliated cells from patients, particularly pediatric patients, presents multiple difficulties. Biopsies and blood samples are invasive, and patients (and their parents) may be reluctant to travel to medical centers, especially for research purposes. We sought to develop non-invasive methods of obtaining viable and ciliated primary cells from ciliopathy patients which could be obtained in the home environment.

**Findings:**

We introduce two methods for the non-invasive acquisition of primary ciliated cells. In one approach, we collected spontaneously shed deciduous (milk) teeth from children. Fibroblast-like cells were observed after approximately 2 weeks of culture of fragmented teeth. Secondly, urine samples were collected from children or adults. Cellular content was isolated and after approximately 1 week, renal epithelial cells were observed. Both urine and tooth-derived cells ciliate and express ciliary proteins visible with immunofluorescence. Urine-derived renal epithelial cells (URECs) are amenable to 3D culturing, siRNA knockdown, and *ex vivo* drug testing.

**Conclusions:**

As evidence continues to accumulate showing that the primary cilium has a central role in development and disease, the need for readily available and ciliated patient cells will increase. Here, we introduce two methods for the non-invasive acquisition of cells with primary cilia. We believe that these cells can be used for further *ex vivo* study of ciliopathies and in the future, for personalized medicine.

## Findings

### Primary cells and ciliopathies

When cilia formation or function is perturbed, any of several dozen associated diseases, collectively known as ciliopathies, can occur. While each ciliopathy disease entity is individually rare, collectively they are common (1 in 300–400 individuals) (1). These genetically heterogeneous diseases can involve one or more organ features, ranging from mild to perinatal lethal phenotypes. Given the wide-ranging function attributed to cilia, it is not surprising that defects in these organelles give rise to a multitude of organ-specific functional defects and pathologies. The different ciliopathies related to non-motile cilia dysfunction often affect renal tissue and are typically diagnosed during childhood (1, 2).

For clinical purposes including diagnosis and intervention, primary patient material is of vital importance. Obtaining patient material from pediatric ciliopathy patients via blood samples or skin biopsies can be traumatic for the patients and their parents/caregivers. Furthermore, patients and parents are often reluctant to travel to a medical center to donate material purely for research. Despite these difficulties, primary ciliated cells from patients are extremely useful in researching ciliopathies and obtaining them may one day be an important part of routine clinical practice. We sought a child-friendly solution to derive valuable patient material that would normally be discarded, without causing the patient any physical or emotional discomfort. Here, we describe our experience [1] isolating renal epithelial cells from regular urine samples, called urine-derived renal epithelial cells (URECs), and [2] harvesting fibroblasts from spontaneously shed deciduous teeth; both cell types have a primary cilium.

### Urine-derived epithelial cells

#### Isolation and expansion

Renal epithelial cells are regularly sloughed off of the renal tubule into the urine. These cells can be collected from the urine and specifically cultured to support proliferation of renal epithelial cells, while suppressing the growth of other cell types present in the urine (e.g., transitional and squamous cells) [3]. Urine was collected from patients and controls within the AGORA study protocol (Aetiologic research into Genetic and Occupational/environmental Risk factors for Anomalies in children; http://www.agoraproject.nl/). The study protocol was approved by the regional Committee on Research Involving Human Subjects, Medisch Ethische Toetsingscommissie of the University Medical Center Utrecht (UMCU), the Netherlands and the parents submitted written informed consent for participation.

Approximately 25–150 ml of mid-stream urine is collected inside of a sterile container. The samples may be processed immediately or stored at 4 °C for up to 4 h, which allows transporting samples to a laboratory facility, as urine collection can be done inside or outside of a medical center, e.g., at the home of the patient. We regularly collect urine samples at the home of patients, because parents are more likely to approve participation in research, and then we transport the samples on ice or cold packs.

**Fig. 1:**
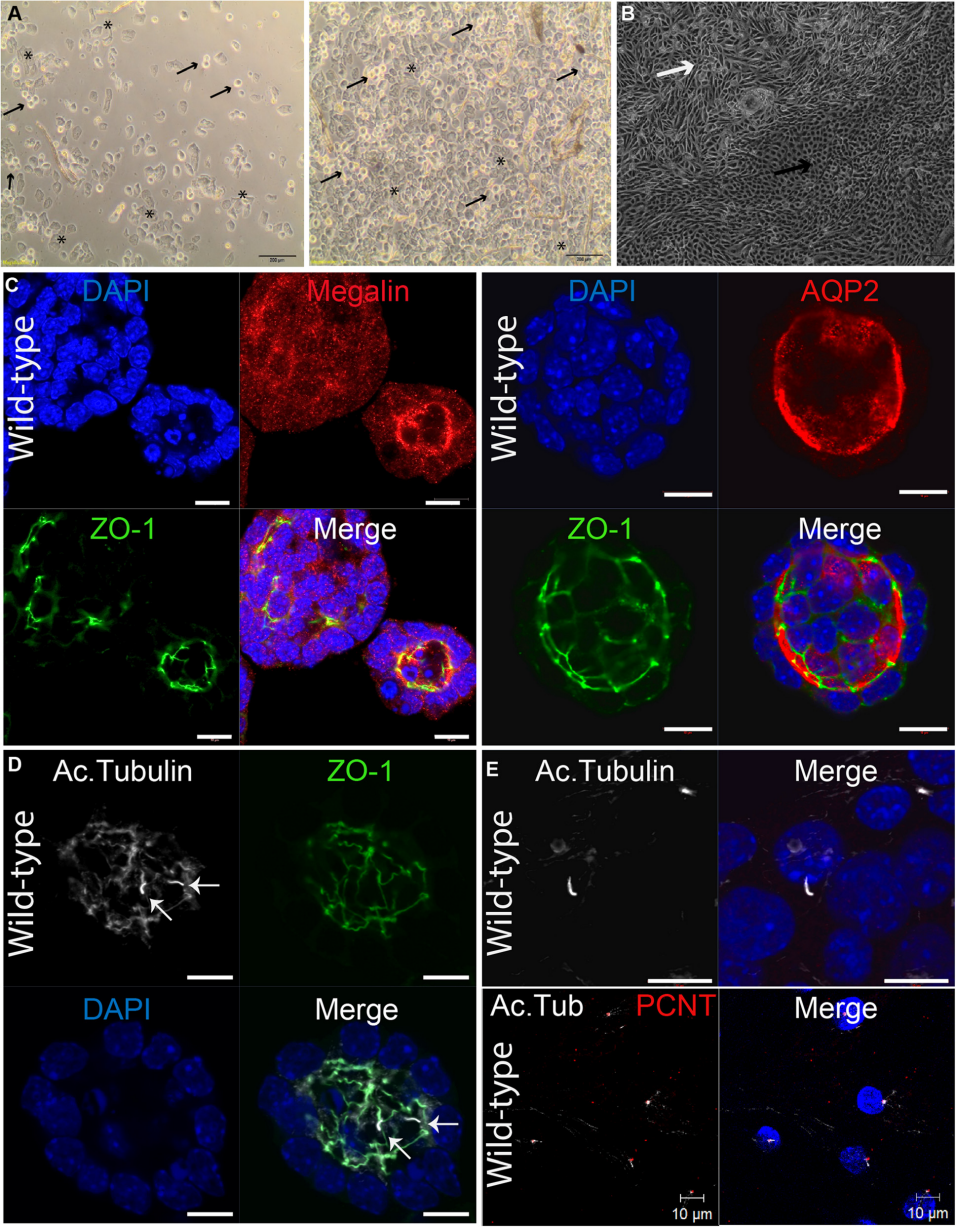
Cultures and images of URECs in 2D and 3D conditions. (**A**) Urine sample 24 h after collection at ×4 magnification. Asterisks indicate squamous cells, and black arrows indicate transitional cells. Scale bar 200 μm. Note that renal epithelial cells are not apparent. (**B**) Renal epithelial cells in culture 12 days after collection at ×4 magnification. Note that there are two morphologically distinct types of renal epithelial cells, marked with a white or black arrow. Scale bar 200 μm. (**C**) Wild-type UREC 3D spheroids. Megalin indicates that cells composing spheroids can be derived from the proximal tubule, while AQP2 was used as a marker for collecting duct cells. Nucleus (DAPI, blue); megalin and AQP2 (red); and ZO-1 (green). Scale bar 10 μm. (**D**) Wild-type UREC 3D spheroids. Cilia are indicated with white arrows. Nucleus (DAPI, blue); Ac. Tubulin (white); and ZO-1 (green). Scale bar 10 μm. (**E**) Wild-type URECs in 2D monolayer. Nucleus (DAPI, blue); Ac. Tubulin (white); pericentrin (PCNT, red). Scale bar 10 μm

Through a series of centrifugation and washing steps, cellular material is isolated and plated on a 24-well plate using renal epithelial growth medium and incubated at 37 °C. Initially, several cell types (squamous and transitional cells) are present, and no URECs are visible (Fig. 1A). Primary medium, which is used to enhance initial adherence and survival, is added for 3 days. After day 3, renal epithelial proliferation medium, which selectively supports renal epithelial proliferation, is used and changed daily. Early UREC colonies become visible 3–15 days after sample collecting and cell isolation. Cells are ready for passaging at 80–90 % confluence around 9–20 days from collection (Fig. 1B).

#### Analysis and current applications

One advantage of URECs is that they can grow in 3D culture to develop spheroids, which are physiologically relevant models of the renal epithelium. After 3–5 days in matrigel, fully formed spheroids develop apicobasal polarity, ciliate, and form complete lumens (Fig. 1C), although we do not observe as many clear lumens as we see in mouse inner medullary collecting duct cells [4]. Such characteristics make these UREC spheroids an excellent approximation of in vivo renal epithelial conditions. The matrigel can be subsequently dissolved and spheroids fixed.

We show that URECs from healthy controls are made up of a mean of 35 % (range 30–40 %, *n* = 2) megalin-positive proximal tubule cells and 57.5 % (range 50–70 %, *n* = 4) aquaporin 2 (AQP2)-positive collecting duct cells (Fig. 1C). We have demonstrated that whereas URECs from healthy individuals ciliate well in both monolayer (mean 54 % ciliated, range 52–56 %, *n* = 2; Fig. 1E) and 3D culture (mean 58.2 % ciliated, range 44–72 %, *n* = 4; Fig. 1D), URECs from Joubert syndrome ciliopathy patients did not (mean 29 % ciliated, range 25–33 %, *n* = 2) [5]. The proportion of ciliated cells is obtained by examining at least 100 nuclei per sample. To date, we have isolated renal epithelial cells from *n* = 50 healthy donors and *n* = 20 from ciliopathy patients. However, several of these samples have become contaminated with bacteria or fungi and several samples have failed to deliver viable cells.

While cultured renal epithelial cells have been extensively used in spheroid models to test gene variants, the use of human URECs in kidney and ciliopathy research offers patient-specific information and the potential to screen for pharmaceutical intervention. For example, we recently showed that URECs from a Joubert syndrome patient with a *CEP290* mutation were grown in spheroids and partially rescued by treatment with a Hedgehog agonist, confirming involvement of the Hedgehog pathway in nephronophthisis in Joubert syndrome [5]. Furthermore, we have observed that these cells grow in a monolayer (Fig. 1E), are amenable to siRNA knockdown (not shown), and can be used for immunofluorescence on cover slips.

### Tooth-derived fibroblast-like cells

#### Isolation and expansion

**Fig. 2:**
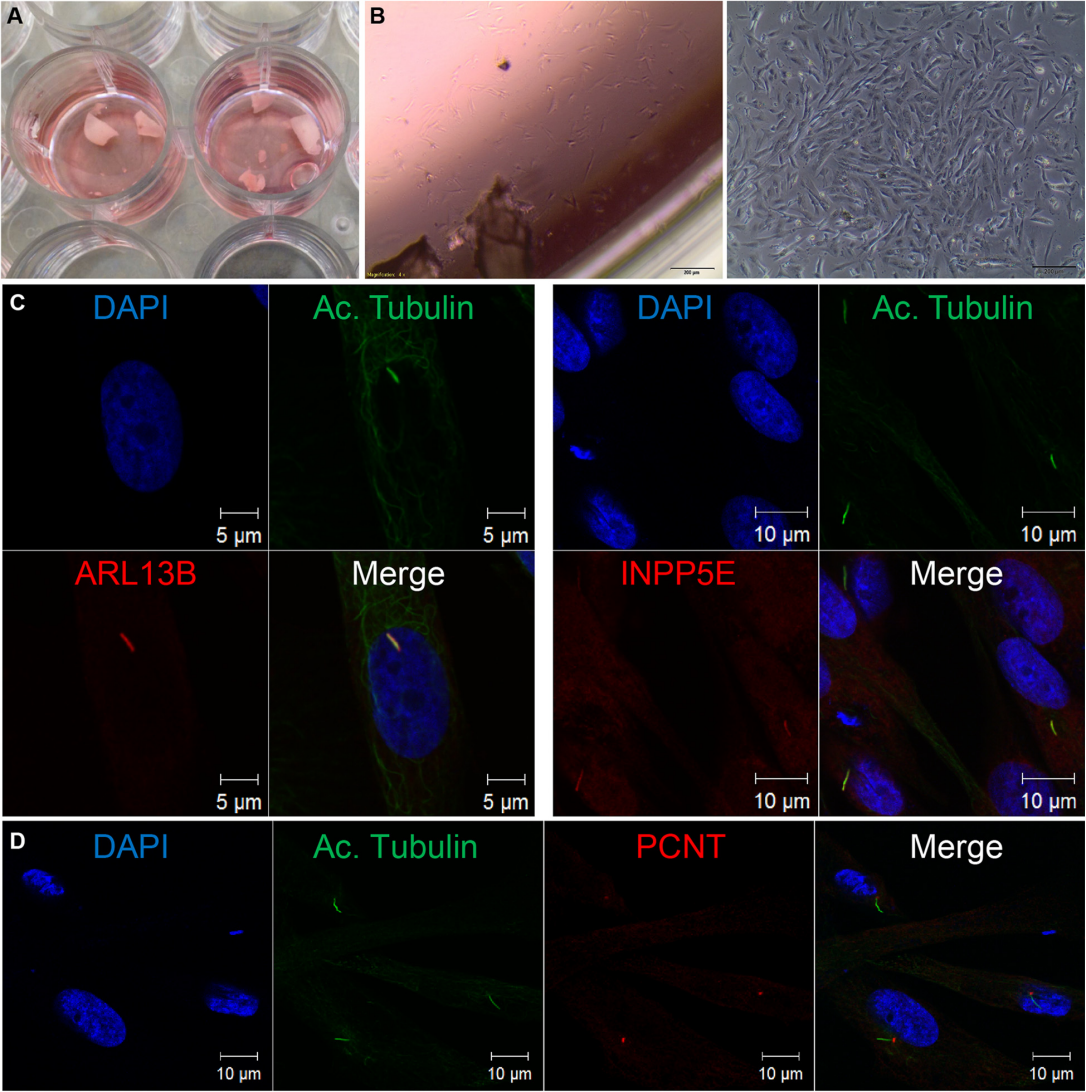
Cultures of cells derived from deciduous teeth. (**A**) Tooth fragments are taken into culture in a 12-well plate under standard fibroblast cell culture conditions. (**B**) Fibroblast-like cells were observed in the culture plate after approximately 2 weeks (left) and expanded ~1 week (right) at ×4 magnification. Scale bar 200 μm. (**C**) Immunofluorescence imaging of fibroblast-like cells from healthy donors (*n* = 3) shows 25–51 % ciliation after 24-h serum starvation. Cells from one donor are shown here. Nucleus (DAPI, blue); ARL13B and INPP5E (red); Ac. Tubulin (green). Scale bar 5 or 10 μm. (**D**). Imaging of fibroblast-like cells from a healthy donor showing cilia and centrosome. Nucleus (DAPI, blue); Ac. Tubulin (green); pericentrin (PCNT; red). Scale bar 10 μm

An alternative non-invasive source of ciliated cells from pediatric ciliopathy patients is spontaneously shed deciduous (milk/baby) teeth shed between the ages of 5 and 14 years. Within 24 h of spontaneous loss of the tooth, the tooth should be kept moist and cool (ideally 4 °C) and transported to the laboratory facility. The PBS-washed enamel casing is then crushed by a hammer in semi-sterile conditions. Tooth fragments are taken into culture under standard fibroblast cell culture conditions and incubated at 37 °C in a 12-well plate in Dulbecco’s Modified Eagle Medium (Fig. 2A). Fibroblast-like cells were observed in the culture plate after approximately 2 weeks. These cells can be frozen and thawed for later use using standard cell culture protocols (Fig. 2B). Teeth were collected from children within the AGORA study protocol approved by the regional Committee on Research Involving Human Subjects. Parents submitted written informed consent for participation.

##### Analysis and current applications

We show that after 24 h of serum starvation, a mean of 41.3 % (range 25–51 % ciliation, *n* = 3, Fig. 2) of the fibroblast-like cells from the tooth of healthy donors ciliate. Common ciliary markers are expressed, such as ADP-ribosylation factor-like protein 13B (ARL13B), inositol polyphosphate-5-phosphatase E (INPP5E) (Fig. 2C) which are associated with Joubert syndrome, as well as centrosome marker pericentrin (PCTN) (Fig. 2D), thus indicating that these cells are an excellent model for investigating ciliary disorders, e.g., Joubert syndrome. To date, we have isolated *n* = 10 healthy fibroblast-like tooth-derived cells; isolation of patient fibroblast-like tooth-derived cells is in progress.

Discarded milk teeth are a child-friendly source of patient material to study ciliopathies or other diseases. The tooth-derived cells can be used for diverse research applications including genomic, epigenetic, or metabolic analysis. Although the timing of the tooth being shed is difficult to plan, the use of milk teeth as a source of material for research is an attractive low-stress option that should be discussed with the parents.

### Discussion

Thus far, we have had promising results in using both tooth- and urine-derived primary ciliated cells for the investigation of human ciliopathies. The usefulness of these methods lies in their non-invasive nature. With these techniques, the pain and inconvenience associated with obtaining blood samples and skin biopsies need not limit the availability of patient material. Although we have used these cells for investigating ciliopathies, they may also be used in other areas where primary cells are needed. Furthermore, their collection and expansion are relatively simple techniques that can be performed by an individual with basic laboratory experience and no specialized equipment.

A common difficulty with the isolation and expansion of primary cells is contamination due to semi-sterile collection, and we have found our techniques to be no exception. Fungal and/or bacterial infections may appear several days after plating. Minimizing the risk of contamination in both cell types involves performing all work in a sterile laminar flow hood and treating prophylactically with antibiotics and antifungal agents in the media. For URECs, ensure that urine is carefully collected mid-stream. The tooth-derived cells require approximately 4 weeks of culturing, from the point of collection until sufficient cells are present for experimentation and freezing. This length of time in culture is not ideal for clinical purposes and also increases the risk of contamination. Another limitation of both techniques is that processing must occur within a specific time frame (4 h for URECs and 24 h for tooth cells). For a more detailed discussion of UREC types seen in culture, see Dorrenhaus et al. [6].

### Conclusion

As evidence continues to accumulate showing that the primary cilium has a central role in development and disease, the need for readily available and ciliated patient cells will increase. Here, we introduce two methods for the non-invasive acquisition of primary patient cells that ciliate well. We believe that these cells can be utilized for further *ex vivo* study of ciliopathies, drug testing, DNA mutation analysis, metabolomics, and functional testing and may be used as sources for the generation of inducible pluripotent stem cells.

## Abbreviations

UREC: urine-derived renal epithelial cell
Ac. Tubulin: acetylated-α-tubulin
AQP2: aquaporin 2
ARL13B: ADP-ribosylation factor-like protein 13B
INPP5E: inositol polyphosphate-5-phosphatases E
PCTN: pericentrin
mIMCD3: mouse inner medullary collecting duct
AGORA: Aetiologic research into Genetic and Occupational/environmental Risk factors for Anomalies in children
